# RETRACTION: Effect of Minimally Invasive Versus Open Surgery in Hepatectomy on Postoperative Wound Complications in Patients With Hepatocellular Carcinoma: A Meta‐Analysis

**DOI:** 10.1111/iwj.70426

**Published:** 2025-03-26

## Abstract

**RETRACTION:** ZhangJ. , ShiM. , DingW. , DuanM. , DaiZ. , and ChenY. , “Effect of Minimally Invasive Versus Open Surgery in Hepatectomy on Postoperative Wound Complications in Patients With Hepatocellular Carcinoma: A Meta‐Analysis,” International Wound Journal 20, no. 10 (2023): 4159–4165, 10.1111/iwj.14313.37442783 PMC10681463

The above article, published online on 13 July 2023, in Wiley Online Library (http://onlinelibrary.wiley.com/), has been retracted by agreement between the journal Editor in Chief, Professor Keith Harding; and John Wiley & Sons Ltd. Following an investigation by the publisher, all parties have concluded that this article was accepted solely on the basis of a compromised peer review process. The editors have therefore decided to retract the article. The authors did not indicate their agreement with the retraction.

**RETRACTION:** J. Zhang , M. Shi , W. Ding , M. Duan , Z. Dai , and Y. Chen , “Effect of Minimally Invasive Versus Open Surgery in Hepatectomy on Postoperative Wound Complications in Patients With Hepatocellular Carcinoma: A Meta‐Analysis,” International Wound Journal 20, no. 10 (2023): 4159–4165, 10.1111/iwj.14313.37442783 PMC10681463

The above article, published online on 13 July 2023, in Wiley Online Library (http://onlinelibrary.wiley.com/), has been retracted by agreement between the journal Editor in Chief, Professor Keith Harding; and John Wiley & Sons Ltd. Following an investigation by the publisher, all parties have concluded that this article was accepted solely on the basis of a compromised peer review process. The editors have therefore decided to retract the article. The authors did not indicate their agreement with the retraction.

